# Facial analytics based on a coordinate extrapolation system (zFACE) for morphometric phenotyping of developing zebrafish

**DOI:** 10.1242/dmm.049868

**Published:** 2023-06-02

**Authors:** Lorena Maili, Oscar E. Ruiz, Philip H. Kahan, Frankie Chiu, Stephen T. Larson, S. Shahrukh Hashmi, Jacqueline T. Hecht, George T. Eisenhoffer

**Affiliations:** ^1^ Pediatric Research Center, Department of Pediatrics, McGovern Medical School University of Texas Health Science Center-Houston, Houston, TX 77030, USA; ^2^ Genetics and Epigenetics Graduate Program, The University of Texas MD Anderson Cancer Center UTHealth Houston Graduate School of Biomedical Sciences, Houston, TX 77030, USA; ^3^ Department of Genetics, The University of Texas MD Anderson Cancer Center, Houston, TX 77030, USA

**Keywords:** Geometric morphometrics, Zebrafish, Craniofacial development, *smarca4a*

## Abstract

Facial development requires a complex and coordinated series of cellular events that, when perturbed, can lead to structural birth defects. A quantitative approach to quickly assess morphological changes could address how genetic or environmental inputs lead to differences in facial shape and promote malformations. Here, we report on a method to rapidly analyze craniofacial development in zebrafish embryos using facial analytics based on a coordinate extrapolation system, termed zFACE. Confocal images capture facial structures and morphometric data are quantified based on anatomical landmarks present during development. The quantitative morphometric data can detect phenotypic variation and inform on changes in facial morphology. We applied this approach to show that loss of *smarca4a* in developing zebrafish leads to craniofacial anomalies, microcephaly and alterations in brain morphology. These changes are characteristic of Coffin–Siris syndrome, a rare human genetic disorder associated with mutations in *SMARCA4*. Multivariate analysis of zFACE data facilitated the classification of *smarca4a* mutants based on changes in specific phenotypic characteristics. Together, zFACE provides a way to rapidly and quantitatively assess the impact of genetic alterations on craniofacial development in zebrafish.

## Teaser

**Summary:** We report a method to rapidly analyze craniofacial development in zebrafish embryos using morphometric analysis, creating a new platform to quantitatively detect phenotypic variation after genetic alteration.

## INTRODUCTION

Vertebrate craniofacial development requires the complex orchestration of cellular processes, molecular signals and interactions between different cell types (Chai and Maxson, 2006; Ji et al., 2020). During the course of organ development, interactions between different tissues are critical in the regulation of cellular proliferation, migration and apoptosis to develop the intricate features that comprise the face (Chai and Maxson, 2006; Compagnucci et al., 2021). Failure to efficiently coordinate the specification, migration, proliferation and apoptosis of cells can lead to craniofacial malformations and structural birth defects, including cleft lip and/or palate, craniosynostosis and facial dysostosis (Hennekam et al., 2010; Van Otterloo et al., 2016). Many developmental syndromes, such as Stickler syndrome, Van der Woude syndrome and Coffin–Siris syndrome (CSS), include craniofacial anomalies in addition to other congenital malformations (Gorlin et al., 2011; Twigg and Wilkie, 2015). One impediment to understanding how genetic variants promote craniofacial anomalies has been our ability to visualize the complex and coordinated cellular interactions during craniofacial development *in vivo*.

Animal models, such as mouse, chicken, frog and zebrafish, provide an important avenue to gain better mechanistic and genetic insights into human craniofacial development (Van Otterloo et al., 2016). Zebrafish offer many advantages as a model system that make them ideal for detailed craniofacial studies – they develop externally and generate large numbers of transparent embryos, which permits unparalleled high-resolution imaging of specific cell types and structures in living vertebrates (Eames et al., 2013; Nichols et al., 2013; Mork and Crump, 2015; Eisenhoffer et al., 2017; Choe et al., 2021; Shull et al., 2022). Zebrafish craniofacial development, particularly craniofacial bone and cartilage formation, has been well characterized and is comparable to amniote craniofacial development. For example, the development of the anterior neurocranium/ethmoid plate is thought to be functionally analogous to palate development in mammals (Swartz et al., 2011; Dougherty et al., 2013). Zebrafish have been used to systematically test the function of genes associated with birth defects in humans (Eberhart et al., 2008; Yuan et al., 2012; Bhatia et al., 2015; Deml et al., 2015; Swindell et al., 2015; Küry et al., 2017; Machado and Eames, 2017; Shaw et al., 2017; Teng et al., 2018; Fernandes and Lovely, 2021; Truong and Artinger, 2021), and an array of conserved genetic variants/mutations already exist that display altered craniofacial development (Amsterdam et al., 2004; McCarthy et al., 2013). Yet, a standardized, unbiased method that can be used to quantitatively assess phenotypic changes in craniofacial structures resulting from these mutations is not currently available.

Geometric morphometrics (GMM) is a quantitative method used to measure and statistically test for variation(s) in shape (Hallgrimsson et al., 2015). GMM methods have been applied to skeletal and soft tissue data to evaluate craniofacial morphology in the clinical setting and to better understand human craniofacial disorders, such as cleft lip and palate, craniosynostosis, ectodermal dysplasia and neurodevelopmental disorders (Weinberg et al., 2009; Goodwin et al., 2014; Cocos and Halazonetis, 2017; Katsadouris and Halazonetis, 2017; Kesterke et al., 2018; Mercan et al., 2020; Rutland et al., 2021). The presentation of craniofacial abnormalities and syndromes often display phenotypic heterogeneity, and the use of GMM provides an important approach that captures critical information that would otherwise be missed using only a discrete categorical variable such as the presence or absence of a normal or abnormal phenotype (Hallgrimsson et al., 2015). For example, GMM has been used to identify facial differences in unaffected relatives of individuals with non-syndromic cleft lip and palate and strain-specific differences in embryonic facial shape underlying susceptibility to developing orofacial clefts in mice (Young et al., 2007; Weinberg et al., 2009).

To leverage the power of the GMM approach for zebrafish embryos, we developed a novel and easily implemented method, facial analytics based on a coordinate extrapolation system (zFACE), to visualize the developing rostral/frontal craniofacial region and analyze quantitative facial morphometric data in a semi-automated way. We applied zFACE to show that loss of *smarca4a* modifies craniofacial morphology in zebrafish embryos and identified regional differences that contribute to the observed altered facial dimensions. These results support *smarca4a* mutant zebrafish as an animal model to provide insight into the craniofacial abnormalities associated with CSS, a rare genetic disorder associated with mutations in *SMARCA4* (*BRG1*). Together, our GMM-based approach and its application demonstrate the power of zFACE to extend our understanding of phenotypic changes in craniofacial development associated with genetic alterations in a vertebrate embryo.

## RESULTS

### zFACE

**Fig. 1.**
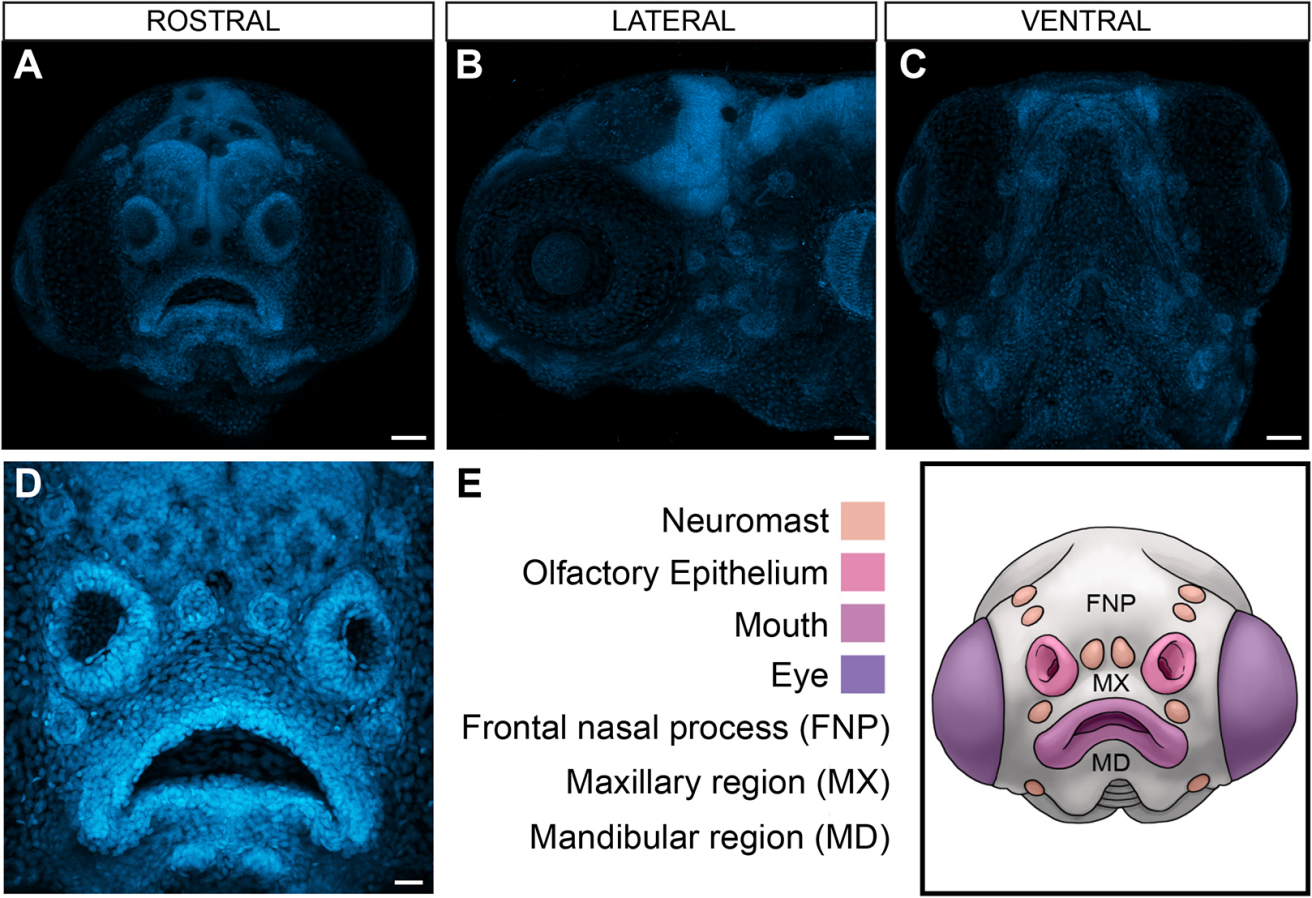
**Craniofacial features visualized in frontal/rostral mounts by DAPI staining and confocal microscopy.** (A-D) Rostral (A,D), lateral (B) and ventral (C) views of developing zebrafish larvae at 5 days post-fertilization (dpf). (E) Schematic representing the facial anatomical structures visualized in the rostral view, including neuromasts, olfactory placodes, eyes, mouth, frontonasal region (FNP), maxillary region (MX) and mandibular region (MD). Scale bars: 50 µm (A-C), 20 µm (D).

Zebrafish embryos develop externally and provide a unique opportunity to visualize development (Mork and Crump, 2015; Eisenhoffer et al., 2017; Machado and Eames, 2017). Although zebrafish craniofacial development has been well characterized, most studies have used lateral and ventral views of the developing face. To visualize additional craniofacial anatomical structures, we first established a simple imaging paradigm using 4′,6-diamidino-2-phenylindole (DAPI), a stain that labels the nucleus of every cell, along with a face-on rostral mounting method using low-melt agarose (Fig. 1A-E). This method can be easily adapted to accommodate different developmental stages, when the orientation of the face with respect to the rest of the body varies, by adjusting the mounting angle of the specimen. However, it is important to mount specimens as consistently as possible in the same orientation for a given developmental stage or mutant. The resulting images capture structures such as the neuromasts, olfactory placodes, eyes, oral cavity, and frontonasal, maxillary and mandibular regions (Fig. 1D,E). Thus, this rostral mounting paradigm provides high-resolution images that reveal important facial information compared to the commonly used lateral and ventral views (Fig. 1B,C).

**Fig. 2.**
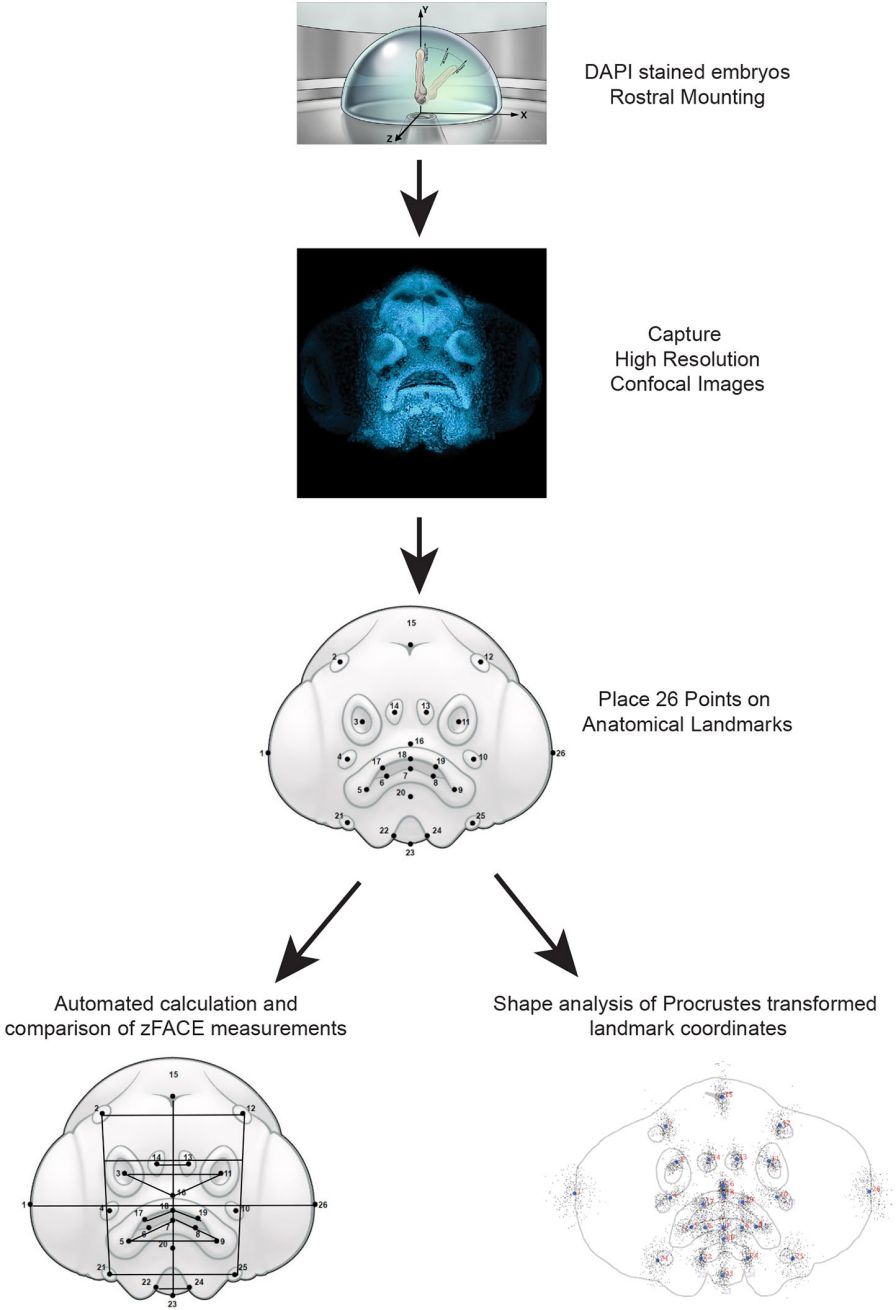
**Facial analytics based on a coordinate extrapolation system (zFACE) workflow for morphometric analysis of the developing zebrafish craniofacial region.** Schematic representing the steps in zFACE analysis, in which DAPI-stained larvae are mounted, images are acquired, points are placed on the 26 anatomical landmarks, and either feature-based zFACE calculations or overall shape analyses can be performed.

We applied GMM to quantitatively analyze facial form based on this newfound ability to identify facial features and landmarks using zFACE. Based on the information captured by the confocal images, 26 easily identifiable landmarks during the embryonic and early larval stages of development were established (Fig. 2; Table S1). Given the complex shape of the oral cavity, and that many craniofacial abnormalities affect the mouth, approximately one-third of the landmarks were assigned to this area to provide better resolution of changes in morphology for this region. Additionally, landmarks were chosen and named to parallel those used in human GMM studies (Weinberg et al., 2009; Liang et al., 2013). Using these established landmarks, an automated calculation of 39 different linear distances, angles and areas was generated to determine the localization of landmarks relative to each other. As repeatability of landmark placement is critical for downstream analysis and comparison, we provide 28 images of 5 days post-fertilization (dpf) wild-type (WT) AB larvae for users to assess consistency among each user, as well as variation between different users (see https://doi.org/10.6084/m9.figshare.22732166.v1) (Klingenberg and McIntyre, 1998; Fruciano, 2016; Špelić et al., 2021; Vrdoljak et al., 2021). Additional statistical approaches were then applied to evaluate overall facial shape (Fig. 2; Table S1). Together, these analyses can be used to quantify phenotypic information resulting from genetic perturbation or environmental exposures and provide valuable information about craniofacial form.

### Morphometric analysis of facial development in zebrafish

**Fig. 3.**
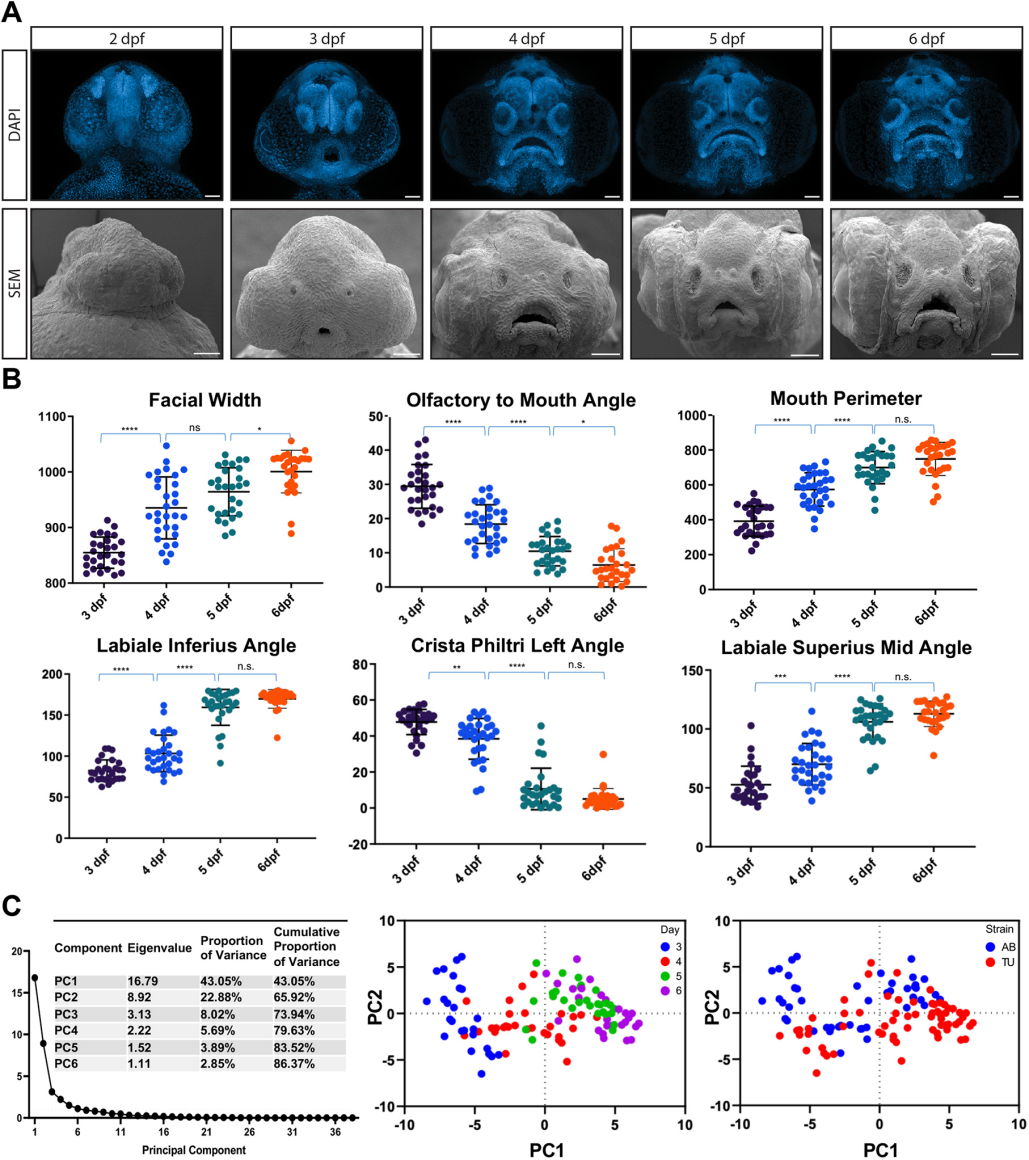
**Time series of zebrafish craniofacial development.** (A) Zebrafish larvae were analyzed from 2 to 6 dpf. Scanning electron microscope (SEM) images confirmed the soft tissue morphology captured by DAPI staining and confocal microscopy used in zFACE. (B) Changes in zFACE features were followed across developmental time points, and alterations in 28 measurements were determined (representative changes are shown in the graphs). The numbers of individual larvae for each group were as follows: *n*=30 at 3 dpf, *n*=31 at 4 dpf, *n*=28 at 5 dpf and *n*=28 at 6 dpf. Specifically, 20 zFACE measurements changed between 3 and 4 dpf, 12 changed between 4 and 5 dpf, and 6 nominally changed between 5 and 6 dpf. *P<0.05, **P<0.01, ***P<0.001, ****P<0.0001; n.s., not significant (one-way ANOVA). (C) Untransformed zFACE features were subjected to multivariate statistical testing using principal component analysis (PCA). The first two components captured 66% of the variance in the dataset. There were differences in developmental times that were captured across PC1, with no strain-specific differences observed. Scale bars: 50 µm.

To assess the ability to detect changes in specific anatomical structures and locations, we used zFACE to investigate facial development over time. Images of rostrally mounted zebrafish embryos from 48 h post-fertilization (hpf) to 6 dpf were acquired by confocal microscopy and compared to scanning electron microscope (SEM) images. The morphology of the soft tissue and anatomical structures was similar in both conditions, indicating that fixation and the DAPI stain, mounting technique or confocal capture does not induce artifacts in facial form (Fig. 3A). The resulting confocal and SEM images revealed the emergence of specific anatomical structures and regional changes that occurred at defined times throughout development. For example, between 2 and 3 dpf, the oral cavity expands into a semi-circle morphology, while the olfactory placodes shift ventrally. Between 3 and 4 dpf, the biggest changes include a narrowing of the face and an enlargement of the mouth, while between 4 and 5 dpf, the midface widens and the oral cavity becomes crescent shaped. Comparison of 5 and 6 dpf confocal images did not discern any major changes in morphology. All of the 26 anatomical landmarks used by zFACE were not present until 3 dpf, and, therefore, quantitative analysis included the course of development from 3 to 6 dpf.

The resulting zFACE measurements were compared across developmental time points and strains. In total, 23 measurements showed a significant change between consecutive developmental day comparisons (3 and 4 dpf, 4 and 5 dpf, or 5 and 6 dpf). The most changes were observed between 3 and 4 dpf, when 20 measurements, including facial width, mouth width and height, olfactory to mouth angles, as well as multiple neuromast measurements, were observed to be significantly different (Table 1, Fig. 3B). Comparison of 4 and 5 dpf measurements revealed 12 significantly different measurements, which included a further increase in mouth width and a decrease in mouth height, as well as more pronounced changes in labiale inferius, crista philtri and labiale superius angles (Table 1, Fig. 3B). Additionally, when later-stage (5 and 6 dpf) larvae were compared, no measurements met the Bonferroni-corrected *P*-value cutoff (six were nominally significant), suggesting that facial morphology was very similar between these two time points (Table 1, Fig. 3B). Interestingly, eight measurements showed a daily increase, ten showed a daily decrease, and five showed a non-linear change (Table 1). Comparison of facial development between the two most commonly used laboratory zebrafish strains, AB and TU, showed relatively few significant differences (Table S2) among the 39 different measurements, suggesting that the strains are very similar in both the timing of anatomical changes and overall facial morphology (Suurväli et al., 2020). This analysis identified time points in developing zebrafish when facial dimensions are most dynamic and ones when face shape remains more stable.

**Table 1.**
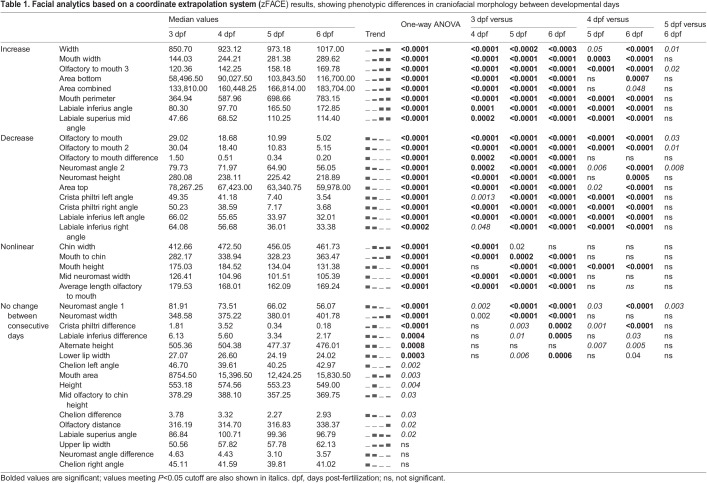
Facial analytics based on a coordinate extrapolation system (zFACE) results, showing phenotypic differences in craniofacial morphology between developmental days

To understand which zFACE measurements contribute the most variance throughout development, we performed multivariate principal component analysis (PCA) of the combined AB and TU groups for all time points. Six principal components met the Kaiser cutoff and collectively accounted for 86% of the variation in the dataset (Fig. 3C). Examination of the principal component (PC) loadings was then used to identify the measurements driving variance along each component. Results showed that neuromast width significantly loaded into PC1, whereas mouth height, mouth area, labiale inferius angle, and left and right crista philtri angles significantly loaded into PC2. The labiale superius and left and right chelion angles significantly loaded into PC3 (Table S2). These data suggested that measurements associated with the morphology of the mouth significantly contributed to the differences between developmental stages. Further, examination of PC plots for the first two components revealed clustering of groups by developmental day along PC1 (Fig. 3C), while no strain-specific clusters were observed. These results indicate that unbiased analyses can also be used to understand how facial morphology changes during early craniofacial development and support the results obtained from the automated zFACE calculations.

We next applied shape analysis in MorphoJ (Klingenberg, 2011) to account for the size differences that are expected to vary between developmental time points. A Procrustes superimposition, which transforms shapes so that they are in maximal superimposition, was applied to the zFACE data in an attempt to remove time-dependent variation due to size, position or rotation, and the PCA was repeated; the first four PCs now cumulatively explained 92% of the variance (the first two PCs explained 81%) (Fig. S1A). Examination of the PC plot for the first two components showed very similar results to the PCA using untransformed data, with developmental days varying across PC1 and no strain-based clusters in the data (Fig. S1A). To focus on how facial shape changed as development progressed, discriminant function analysis (DFA) was utilized, and results were summarized by overlaid wireframe representations (Fig. S1B-D). This analysis revealed significant shape changes between 3 and 4 dpf (Procrustes distance=0.14, *P*<0.0001) and 4 and 5 dpf (Procrustes distance=0.12, *P*=0.005), but no changes between 5 and 6 dpf (Procrustes distance=0.05, *P*=0.04 before Bonferroni correction) (Fig. S1B-D). No strain-specific shape differences were observed at any of the developmental time points (Fig. S1E-G). Together, results from these analyses suggest that zFACE represents a robust and sensitive approach for morphometric analysis of facial development in zebrafish embryos.

### Application of zFACE to *smarca4a* mutant zebrafish reveals similarities with CSS

**Fig. 4.**
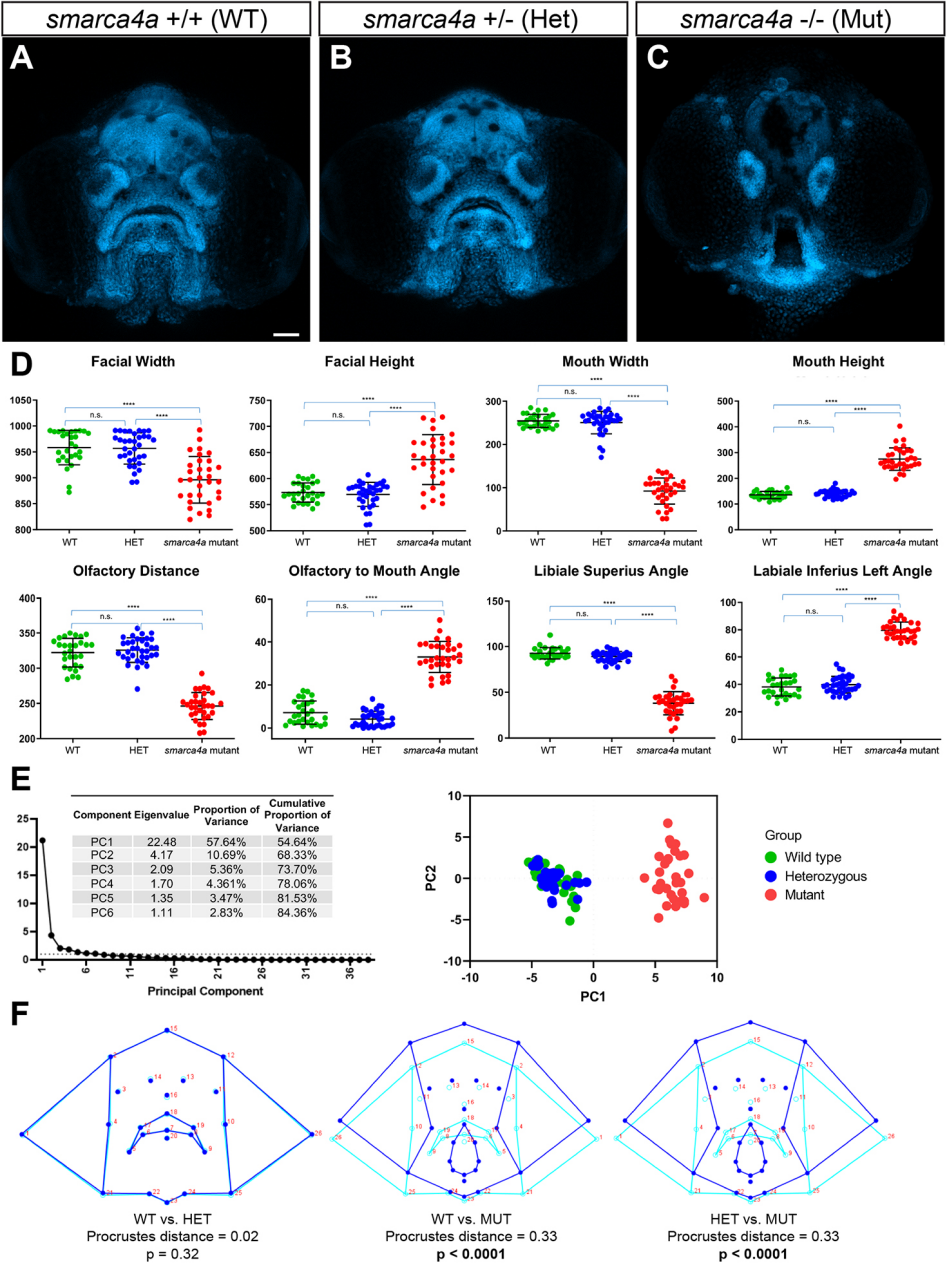
**zFACE analysis of zebrafish with loss of *smarca4a*.** (A-C) Confocal images of wild-type (WT; A), heterozygous (Het; B) and homozygous mutant (Mut; C) *smarca4a* larvae. (D) zFACE analysis pinpointed 33 significantly altered facial measurements between the groups, with examples of dimensions that were unaltered, decreased and increased shown. The numbers of larvae analyzed for each group were *n*=28 for WT, *n*=34 for Het and *n*=32 for Mut. ****P<0.0001; n.s., not significant (one-way ANOVA). (E) Untransformed zFACE features were subjected to multivariate statistical testing using PCA. The first two components explained 68% of the variance in the dataset, and the principal component (PC) plot showed clear separation of the homozygous mutant group from the WT and heterozygote groups. (F) Discriminant function analysis after landmark data were Procrustes transformed further supported that *smarca4a* homozygous mutants had different average facial shapes compared to either WT or heterozygous larvae, whereas WT and heterozygous groups had the same facial shape. Wireframe representations of facial shapes highlight the alterations to landmarks in the upper face, eyes, oral cavity, midface and lower jaw in the homozygous *smarca4a* mutants [discriminant function analysis (DFA)]. Scale bar: 50 µm.

To test the application of zFACE and assess its utility for detection of variation in morphology that occurs after genetic perturbation, we analyzed zebrafish with loss of *smarca4a^a8^*, which have been described to have craniofacial anomalies (Link et al., 2000; Gregg et al., 2003; Eroglu et al., 2006). Our rostral confocal images revealed a severely affected facial phenotype in *smarca4a* homozygous mutant larvae at 5 dpf, whereas heterozygotes and WT larvae showed normal developmental hallmarks (Fig. 4A-C; Fig. S2). *smarca4a* mutants had a narrow head and face, smaller brain, reduced olfactory pits, open and elongated oral cavities, and small mandibles (Fig. 4C). Automated calculation and comparison of zFACE measurements showed that homozygous mutants significantly differed from heterozygotes and WT controls in 33 out of the 39 zFACE measurements, including reduced facial width, increased facial height, decreased olfactory distance, reduced mouth width and altered oral cavity angles (Fig. 4D; one-way ANOVA, post hoc Tukey's test, *P*<0.0013 for all; Table 2). All three groups were equal in upper lip width, chin width, mid olfactory to chin height, mouth area, and the difference between chelion and labiale inferius left and right angles, suggesting that *smarca4a* does not affect these structures.

**Table 2.**
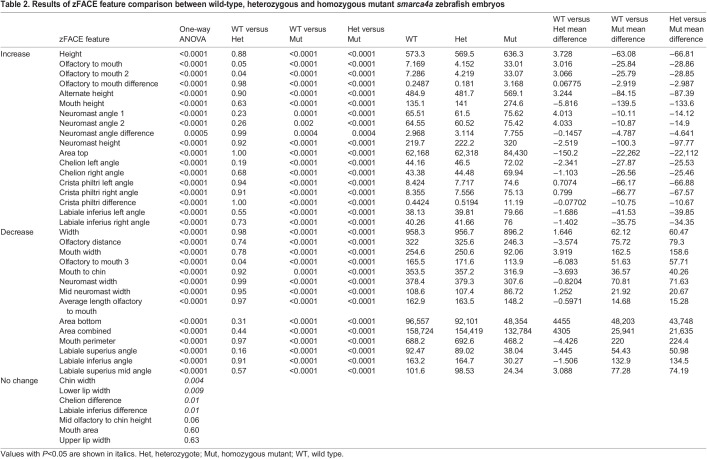
Results of zFACE feature comparison between wild-type, heterozygous and homozygous mutant *smarca4a* zebrafish embryos

Dimensionality reduction via multivariate PCA of zFACE measurements identified six principal components that met the Kaiser cutoff and cumulatively explained 84% of the variance (Fig. 4E). The first component (PC1) explained 58% of the variance, PC2 explained an additional 11%, and PC3 explained 5%. After varimax rotation, component loadings showed that the measurements neuromast height and area top significantly loaded into PC1, whereas width, neuromast width, average length of olfactory to mouth, and facial area combined loaded into PC2. The score plot (PC plot) for these first two components showed *smarca4a* homozygous mutants as having higher PC1 scores and separate from the WT and heterozygous groups (Fig. 4E). Using the PCA model, PC scores were predicted for each embryo, and logistic regression was performed with these predicted scores using group as the dependent variable and PC score as the independent variable. Importantly, PC1 score alone could predict whether an embryo was a homozygous *smarca4a* mutant (when PC1 score was greater than −0.98) but could not distinguish between WT or heterozygote embryos (*P*=0.86) (Fig. 4E). Because the zFACE measurements are composed of different types of units (angles, areas, distances and differences) and the standard deviation (s.d.) between the measurements is not equal, PCA was also performed after standardizing the data and scaling it to have a mean of 0 and s.d. of 1. This led to very similar PCA results, with six PCs cumulatively explaining 88% of the variability. Additionally, we compared 4 and 6 dpf WT embryos (1 day earlier and 1 day later) to the *smarca4a* embryos to examine whether the morphological differences in the mutants could be due to delayed facial development. In the PCA plot, all WT embryos plotted separately from the *smarca4a* homozygous mutants, suggesting that development is not simply delayed and that the abnormal facial phenotype is specific to *smarca4a* disruption (Fig. S3).

Lastly, we performed Procrustes superimposition of the landmark coordinates in MorphoJ (Klingenberg, 2011). Data were analyzed based on the assumption of object symmetry in the head, and the symmetric portion was evaluated in all analyses (Klingenberg et al., 2002). PCA resulted in PC1 accounting for 85% of the variance, PC2 4% and PC3 another 3%; cumulatively these first three PCs explained 92% of the variance (Fig. S4A). Similar to results from the untransformed PCA score plot, the genotype groups could be clearly distinguished by graphing PC1 versus PC2 (Fig. S4B). Shape changes across PC1 affected mouth sphericity, with landmarks around the oral cavity showing the biggest changes (highest eigenvectors); changes across PC2 are suggestive of a narrower midface, and elongated head and mouth (Fig. S4C).

To compare the three genotype groups in an unbiased manner, we used canonical variate analysis (CVA) as an exploratory method. The results showed significant Mahalanobis and Procrustes distance differences among groups, with the *smarca4a* homozygotes significantly differing from the heterozygote and WT groups (Mahalanobis distance=19.85 and Procrustes distance=0.33; *P*<0.0001 for both when homozygotes were compared to WT; and Mahalanobis distance=19.22 and Procrustes distance=0.33; *P*<0.0001 for both when homozygotes were compared to heterozygotes). Changes in canonical variate 1 (CV1)-involved landmarks around the mouth led to a more round and open mouth, whereas those along CV2 were associated with a narrower and elongated face, similar to the results obtained from the PCA analysis (Fig. S4). Because the WT and heterozygous groups were so similar, DFA, which is similar to CVA but compares only two groups at a time, was performed to focus on the shape changes specific to the homozygous mutants. Results showed a Procrustes distance of 0.33 (*P*<0.0001) in both the WT and heterozygote to homozygous mutant comparisons (Fig. 4F), and wireframe representations depicted shape change involving a vertically elongated mouth (Fig. 4F), reflecting the phenotype seen in the confocal images (Fig. 4C; Fig. S2).

**Fig. 5.**
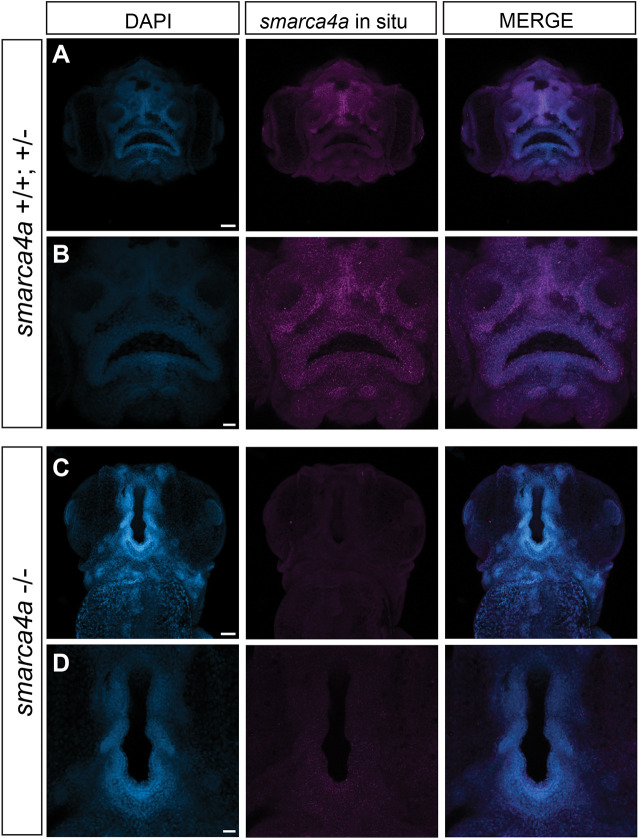
***smarca4a* mRNA expression at 5 dpf.** (A-D) Fluorescent *in situ* hybridization showed that *smarca4a* mRNA is expressed in multiple facial tissues and underlying brain structures in WT and heterozygous zebrafish (A,B), whereas s*marca4a^−^*^/−^ mutants showed dramatically reduced *smarca4a* mRNA expression (C,D). Scale bars: 50 µm (A,C), 20 µm (B,D).

To better understand the observed phenotypic abnormalities, we performed fluorescent *in situ* hybridization to examine *smarca4a* mRNA expression in the craniofacial region. Expression of *smarca4a* mRNA was observed at 5 dpf in several facial tissues, including the perioral and oral tissues, olfactory placodes and the telencephalon in both WT and heterozygous larvae (Fig. 5A,B). *smarca4a^−/−^* mutants, however, showed a dramatically reduced signal in the entire craniofacial region, suggesting that the point mutation (which creates a premature stop codon) leads to no detectable mRNA expression in developing facial structures (Fig. 5C,D) (Gregg et al., 2003). This expression pattern of *smarca4a* mRNA was consistent with the facial structures that zFACE identified as the most altered in the *smarca4a* mutants. Based on the observed morphological changes in brain and neural tissues, the width and length of the brain were measured, and mutants showed an increased length-to-width ratio (one-way ANOVA, *P*<0.0001) (Fig. S5). Together, these results suggest similarities between *smarca4a* mutants and individuals with CSS, who often present with craniofacial abnormalities such as a smaller mouth, thicker lips and reduced philtrum, as well as intellectual disability and microcephaly (Coffin and Siris, 1985; Tsurusaki et al., 2012).

## DISCUSSION

GMM is a powerful quantitative approach for assessing phenotypic differences arising from alterations in shape and size. Here, we developed a streamlined method called zFACE to visualize craniofacial structures and applied GMM to evaluate the developing zebrafish orofacial region. Although similar methods exist for other model organisms and human studies, there have been few applications for zebrafish, and zFACE fills this gap in a way that builds on existing approaches and facilitates cross-species comparisons. We first used zFACE to characterize and understand changes in facial morphology from days 3 to 6 of zebrafish development and established standards at various time points. Additionally, we applied it to analyze *smarca4a^a8^* (*yng*) mutants and uncovered morphometric alterations that coincided with where *smarca4a* was expressed, supporting the use of these mutants to inform on mechanisms driving CSS. Collectively, the development and testing of zFACE presented here supports its use as a powerful quantitative tool to uncover previously unappreciated craniofacial alterations in zebrafish genetic models.

To facilitate widespread use for future studies, we added several key features to make zFACE informative, reliable and easy to implement. A detailed protocol is included at https://doi.org/10.6084/m9.figshare.22732166.v1, along with recommendations for ensuring reproducible capture of structures when imaging and placing landmarks. Template files that automatically calculate measurements, perform basic analyses and plot results from user data are available at https://doi.org/10.6084/m9.figshare.22732166.v1. This feature-focused exploration is useful for quickly identifying regions or structures affected by the experimental variable. For example, reduced size can reflect reduced growth during development and point to possible cell population deficiencies driving effects in a particular region, or globally (Juriloff et al., 2004; Young et al., 2007). The ability to perform shape analysis from the same landmarks in MorphoJ (Klingenberg, 2011), a widely used and open-source morphometric program, offers additional advantages: it is reliable, user friendly, and has been implemented in craniofacial studies in both zebrafish and humans.

Previous studies have relied on lateral and ventral views of zebrafish embryos and larvae, preventing acquisition of information on important aspects of facial morphology. The staining and mounting technique presented here is able to facilitate acquisition of rostral view images. Using this view, standard anatomical locations and nomenclature facilitate comparison with human, mouse and other model organism studies (Young et al., 2007; Weinberg et al., 2008; Dickinson, 2016). However, zFACE is also amendable for expansion to analyze the lateral and ventral views with the selection of custom landmarks that capture relevant structures in these views (Fig. S6). Some challenges for implementing custom landmarks include the variability of new anatomical structures as landmarks, as well as the consideration for mounting angle variation and developmental differences that might be present for the structures of interest. Embryonic/larval zebrafish offer a unique opportunity for detection of small effects due to high-quality imaging capabilities and large numbers of individuals that can be assessed for each condition, providing high-throughput, adequately powered studies. Additionally, we selected a moderate number of landmarks to allow for exploratory analyses such as CVA, for which the number of individuals (*n*) in each group needs to be higher than the number of landmarks (Hallgrimsson et al., 2015). We also designated a sufficient proportion of the landmarks around the mouth to capture the complex and dynamic shape this structure has during the course of development. Lastly, it is important to point out that more advanced analyses might be warranted for some datasets, and the resulting zFACE data can be examined in software packages such as Geomorph or Morpho in R for more complex statistics (Schlager, 2017). Although zFACE handles multiple testing using the Procrustes-based approaches, Euclidean distance matrix analysis, which represents each specimen as a matrix of linear distances between all possible pairs of landmarks and has the ability to detect variation in specific structures or around specific landmarks, can also be utilized (Hallgrimsson et al., 2015).

Here, we applied zFACE to test whether we could detect phenotypic changes after genetic manipulation. Using a mutant with previously reported craniofacial abnormalities but analyzed using only lateral and ventral views, our analysis identified several altered facial features in *smarca4a* mutant larvae that contribute to a different overall average face shape compared to that of WT and heterozygous animals. The abnormal facial features, together with the alterations in brain morphology, show parallels to those observed in patients with CSS. CSS is a rare congenital disorder that presents with distinct facial features due to craniofacial abnormalities, fifth digit anomalies, microcephaly and intellectual disability (Kosho et al., 2014; Mardinian et al., 2021). The clinical features are heterogeneous, and variants in several genes encoding proteins in the SWI/SNF complex, including *SMARCA4A*, have been identified as causal, with variants spanning the gene and disrupting several protein domains [Kosho et al., 2014; Online Mendelian Inheritance in Man (OMIM) #614609]. To date, there are no established animal models for the subtype of CSS with *SMARCA4* mutations (Celen et al., 2017; Filatova et al., 2019). Thus, the *smarca4a* zebrafish larvae provide an opportunity for future studies to better understand disruptions in development and identify potential strategies for therapeutic intervention.

The zebrafish *smarca4a^a8^* mutant, has a point mutation (C-to-A transversion) leading to a premature stop codon early in the gene, preceding all functional domains (Gregg et al., 2003). This mutant has been described as having craniofacial abnormalities, alterations in brain size and patterning and other anomalies in neural crest cell-derived tissues (Eroglu et al., 2006). The current study provides high-resolution phenotypic information to further support these abnormal craniofacial characteristics of *smarca4a* mutants compared to WT and heterozygous controls. Our results identified important similarities in craniofacial features of the mutant with abnormalities reported for CSS. Our results also found severely abnormal oral cavities, suggesting compromised oropharynx function and ability to feed, which is possibly another reason why these mutants only survive to 7 dpf (Link et al., 2000). The frontal view additionally allowed brain measurements of the telencephalon, which showed an altered length-to-width axis, supporting that mutant *smarca4a* leads to abnormal brain morphology (Eroglu et al., 2006). Future studies can use a similar approach to integrate phenotypic data with genetic and molecular information for greater mechanistic insights on gene functions during development.

The complexity of craniofacial development and the structures that make up the craniofacial regions make the detection of subtle phenotypic variation challenging, requiring advanced analytical tools. zFACE can be implemented in a way that is sufficiently powered to detect subtle morphological changes and better allow for genotype–phenotype correlations, which can serve an important role in the investigation of multifactorial disorders, gene–environment interactions, pharmacological interventions and other studies. Direct comparisons on how morphology changes based on genetic perturbations can also bridge the gap between results from genome-wide association studies and the biological effects of genetic variation on craniofacial structures. In conclusion, zFACE can facilitate important studies to examine and integrate the morphological effects of genetic, environmental or developmental perturbations in zebrafish studies of craniofacial development.

## MATERIALS AND METHODS

### Experimental model and subject details

Experiments were conducted on larval zebrafish (*Danio rerio*) maintained under standard laboratory conditions with a cycle of 14 h of light and 10 h of darkness. Larvae were collected and kept in E3 larva medium at 28.5°C and staged as previously described (Westerfield, 1993). The zebrafish used in this study were handled in accordance with the guidelines of the University of Texas MD Anderson Cancer Center Institutional Animal Care and Use Committee and UTHealth Animal Welfare Committee. *smarca4a^a8^* (*yng*) mutant zebrafish (Gregg et al., 2003) were obtained from the Zebrafish International Resource Center.

### Specimen collection, staining, mounting and image acquisition

Specimens were collected and fixed overnight on a shaker in 4% fixation solution prepared from dilution of a 36.5% formaldehyde stock solution (formalin; Sigma-Aldrich) into PBS with a small amount of detergent (0.05% Triton-X 100) (0.05% PBST).

Fixation solution was then removed, the specimens were briefly washed with PBST, and DAPI (Thermo Fisher Scientific) was added to PBST at a 1:1000 dilution. The samples were incubated in the DAPI solution for 30 min and rinsed with 0.05% PBST in a series of three 20 min washes. Samples were then rinsed and stored in PBS at 4°C. Each embryo was mounted in a 35 mm glass-bottom culture dish with a 10 mm microwell (MatTek Corporation) filled with 1% low-melt agarose solution. For the frontal/rostral orientation, each embryo was manipulated by moving the tail to suspend the sample upside down, ensuring that the eyes were on the same plane to reduce variability in mounting angles.

To ensure proper orientation of each sample, the clear visualization of the midline between the brain ventricles and the ability to see folds in lower jaw tissues was used as a guide. If the size of the embryo head was too big, the stage was rotated 45° during image acquisition so both eye lenses could be captured, and the last *z*-stack ensured inclusion of these structures. Typically, the 10× (magnification) objective was used to capture 40 slices of 3.08 μm thickness at 2% laser power using 600 V, digital offset of 2 and digital gain of 1. The images were 1024×1024 at 8 bit/pixel, and the maximum scan speed and averaging of 2 was used. The same magnification was used for all images within a given dataset, and landmarking was performed by the same user.

### Coordinate point system

A 26-point system was utilized for landmarks in the craniofacial region of zebrafish larvae. Each point was assigned a number 1 through 26. Confocal images were opened in ImageJ, points were selected in numerical order using the Point Picker tool, and *xy* coordinates for each point were extracted.

### Facial dimension measurements

Using these *xy* coordinates and the distance formula
(1)

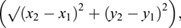
various distance, angular and area measurements were calculated between landmarks. Angular measurements utilized the formula
(2)

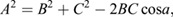
where *a* is the angle of the *BC* connecting point. Triangular areas were found using the formula
(3)

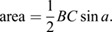
All calculations were performed using Microsoft Excel.

### Statistical analysis of zFACE measurements

A minimum of 28 images were analyzed per stage/condition to provide adequate power for statistical comparison. For statistical analysis of each zFACE measurement, the GraphPad Prism analysis function was used to run one-way ANOVA with Tukey's post hoc tests for multiple comparisons. Bonferroni correction for 39 tests was applied, and *P*<0.00128 was considered significant.

Multivariate analysis of the zFACE measurements was performed in Stata (StataCorp) and GraphPad Prism. Data were standardized and PCA was performed. Multiple models were run to thoroughly identify/evaluate the most robust model in Stata, in which data were rotated and PC loadings could be calculated. Once modeling was determined in Stata, a rapid and streamlined analysis was rerun in GraphPad Prism, producing similar results.

### Shape analysis of zFACE landmark coordinates

Landmark coordinates were imported into MorphoJ, and standard protocols were utilized to perform Procrustes superimposition on principal axes, as well as PCA, CVA and DFA to compare shape changes across the dataset and between groups (Klingenberg, 2011).

### Genotyping of *smarca4a* mutants

*smarca4a^a8^* (*yng*) mutant zebrafish (Gregg et al., 2003) were identified by a PCR reaction using the following primer sequences: Forward 5′-CCTGTCATGCCCCCTCAGAC-3′; and Reverse 5′-CCGACCCCCACTTTGAGAAC-3′. The resulting 190 bp band was excised, and a restriction digest using RsaI was performed at 37°C for 4 h. RsaI only cuts the WT band, resulting in the WT larvae having two bands (50+140 bp), heterozygous larvae having three bands (50+140+190 bp) and homozygous mutants having one 190 bp band.

## Supplementary Material

10.1242/dmm.049868_sup1 Supplementary information
