# Supplementary material for: Facial analytics based on a coordinate extrapolation system (zFACE) for morphometric phenotyping of developing zebrafish
Source: Dis Model Mech. 2023 Jun 2;16(6):dmm049868. doi: 10.1242/dmm.049868 (PMC10245138; doi:10.1242/dmm.049868)
Supplement: Supplementary information [file dmm-16-049868-s1.pdf]

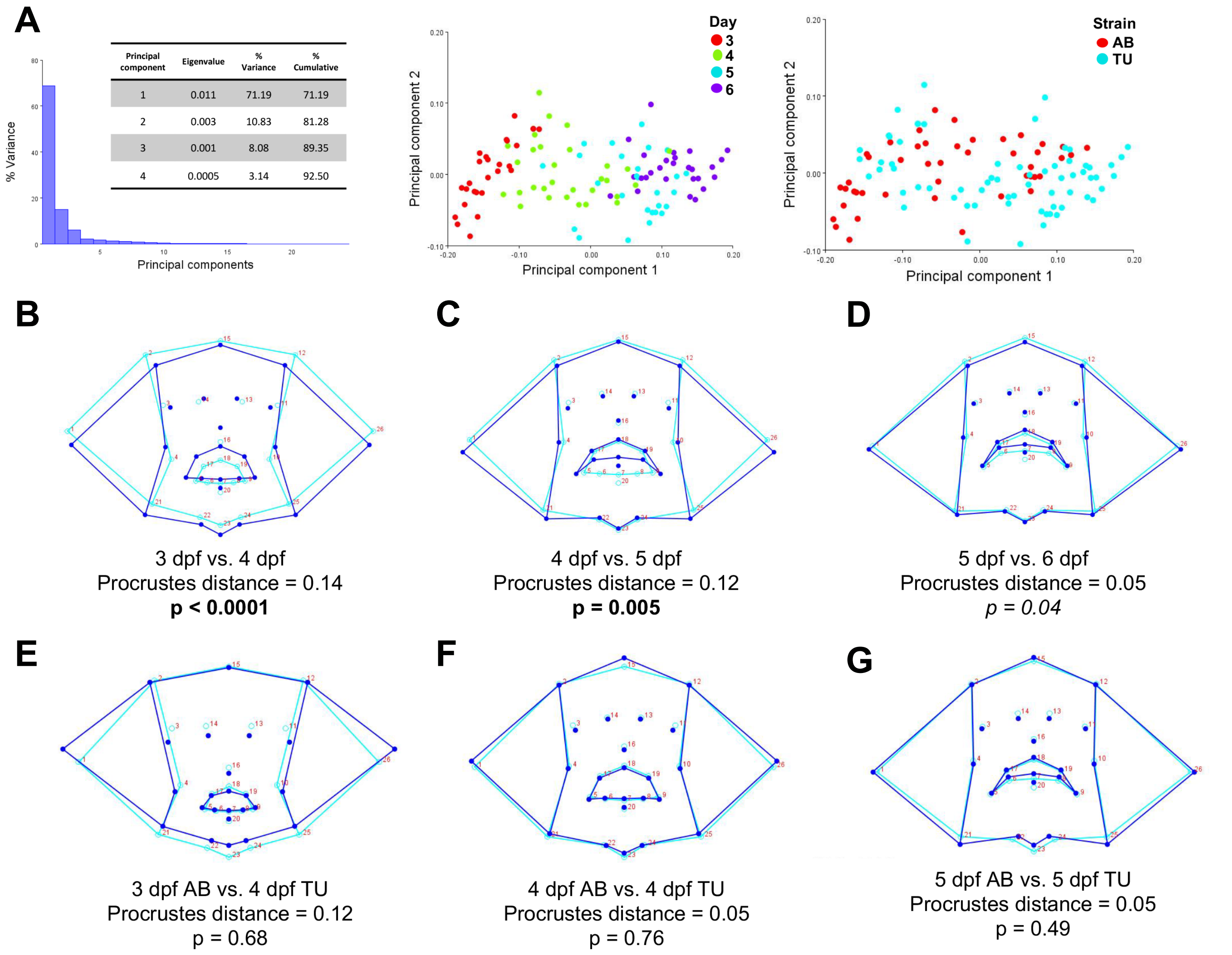

Principal component	Eigenvalue	% Variance	% Cumulative
1	0.011	71.19	71.19
2	0.003	10.83	81.28
3	0.001	8.08	89.35
4	0.0005	3.14	92.50

Fig. S1. Shape analysis using zFACE landmark coordinates. A. PCA was performed after Procrustes superimposition of the zFACE landmark coordinates. The first 2 components explained 81% of the variance in the dataset, and PC plots showed similar clustering of data by developmental day across PC1, while no strain-specific clustering was observed. **B-D.** Discriminant function analysis (DFA) was utilized to identify and follow shape differences between developmental days and between strains. Significant shape changes were found between 3 and 4 dpf and 4 and 5 dpf, while face shape was not different between 5 and 6 dpf. **E-F.** There were no strain-specific differences.

Disease Models & Mechanisms • Supplementary information

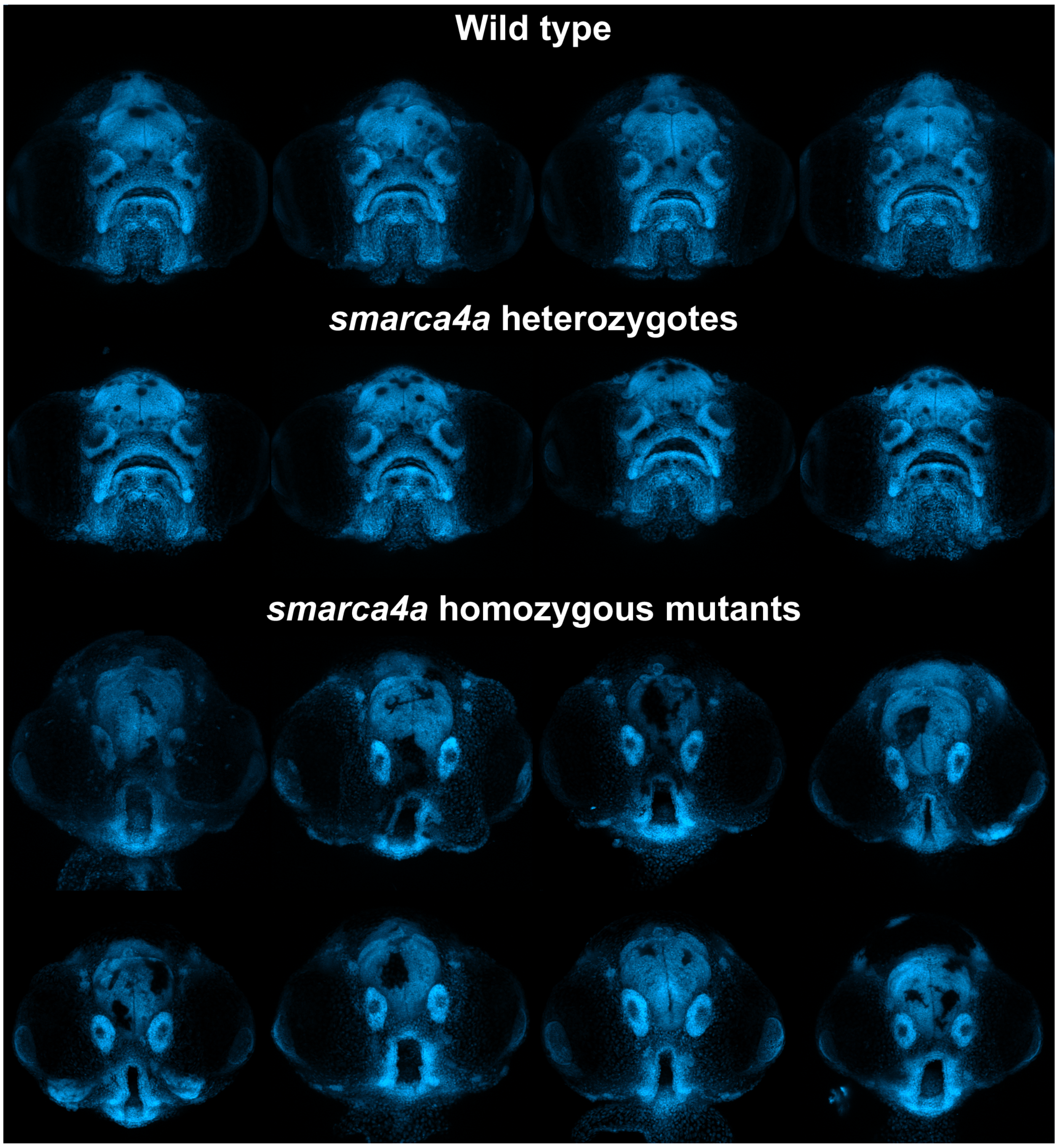

Fig. S2. Phenotype of *smarca4a* mutant larvae. Facial phenotypes for wild-type, heterozygous and *smarca4a* homozygous mutant larvae at 5dpf.

Disease Models & Mechanisms • Supplementary information

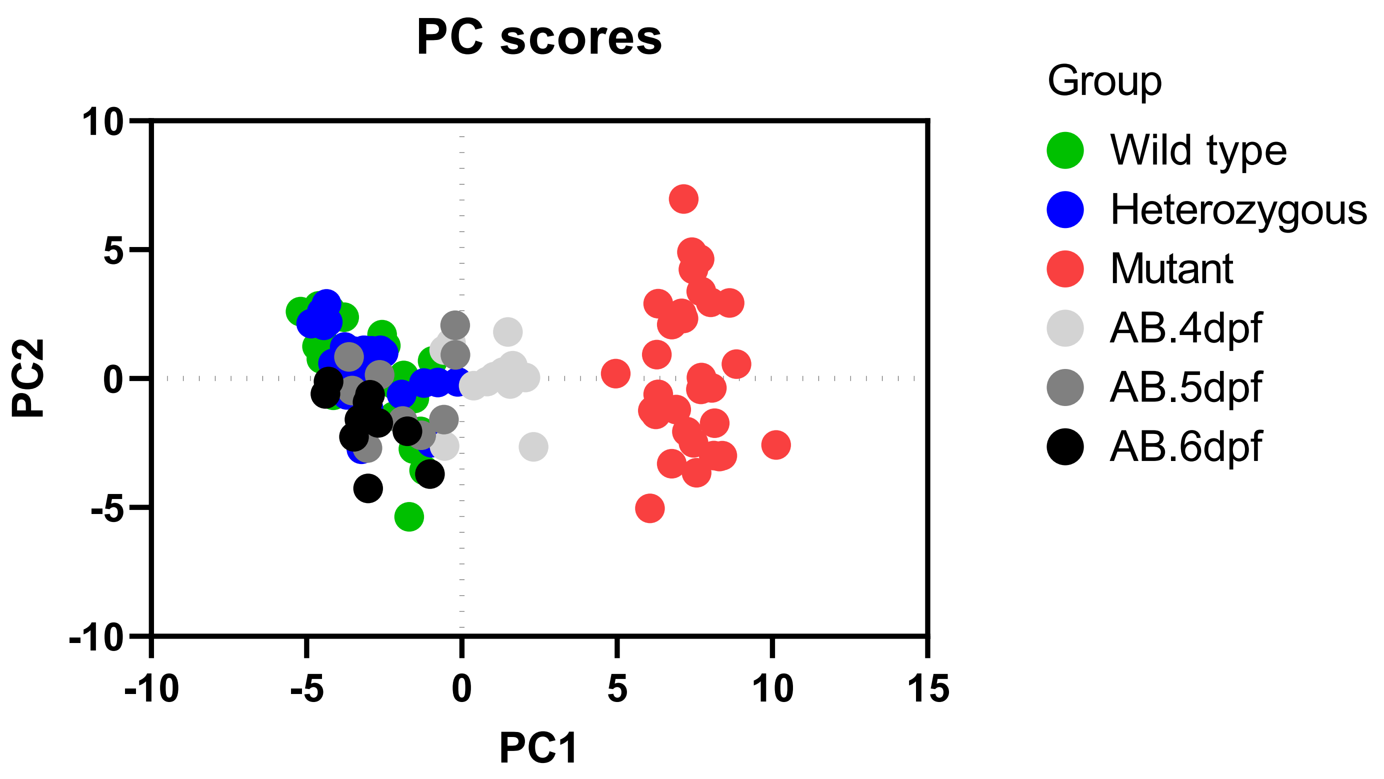

Fig. S3. Comparison of *smarca4a* mutants with other developmental timepoints. PCA model showing that the facial phenotype of *smarca4a* homozygous mutants at 5dpf is different from 4 and 6dpf wild-type larvae.

Disease Models & Mechanisms • Supplementary information

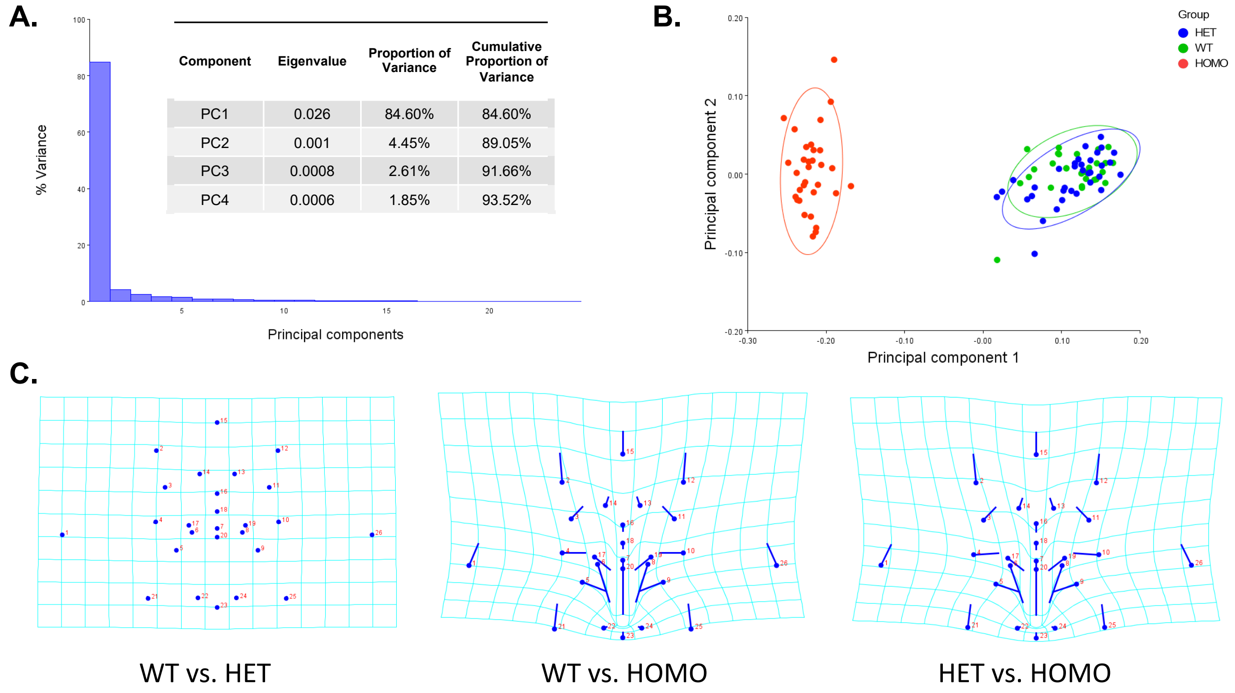

Fig. S4. Analysis of *smarca4a* mutant zebrafish larvae. A. PCA results after Procrustes superimposition. **B.** The component plot with confidence ellipses shows overlap of the wild type and heterozygous *smarca4a* larvae and clear separation of the homozygous mutants from the other two groups. **C.** Transformation grids with lollipop graphs showing the resulting facial shape changes in DFA of *smarca4a* wild type, heterozygous and homozygous mutant larvae.

Disease Models & Mechanisms • Supplementary information

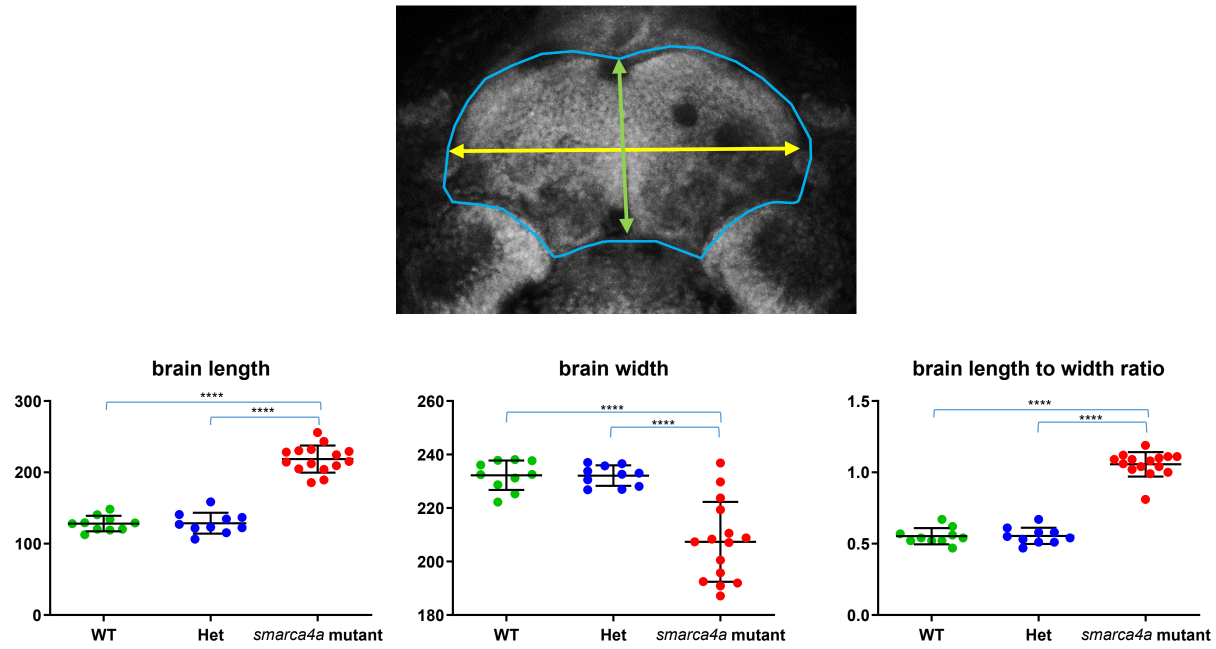

Fig. S5. Altered brain morphology in *smarca4a* mutants. Brain length and width were measured for all genotype groups on the rostral confocal images. S*marca4a* homozygous mutants showed increased length to width ratio compared to wild type and heterozygote larvae, indicating morphological changes in the telencephalon. **** $p < 0.0001$.

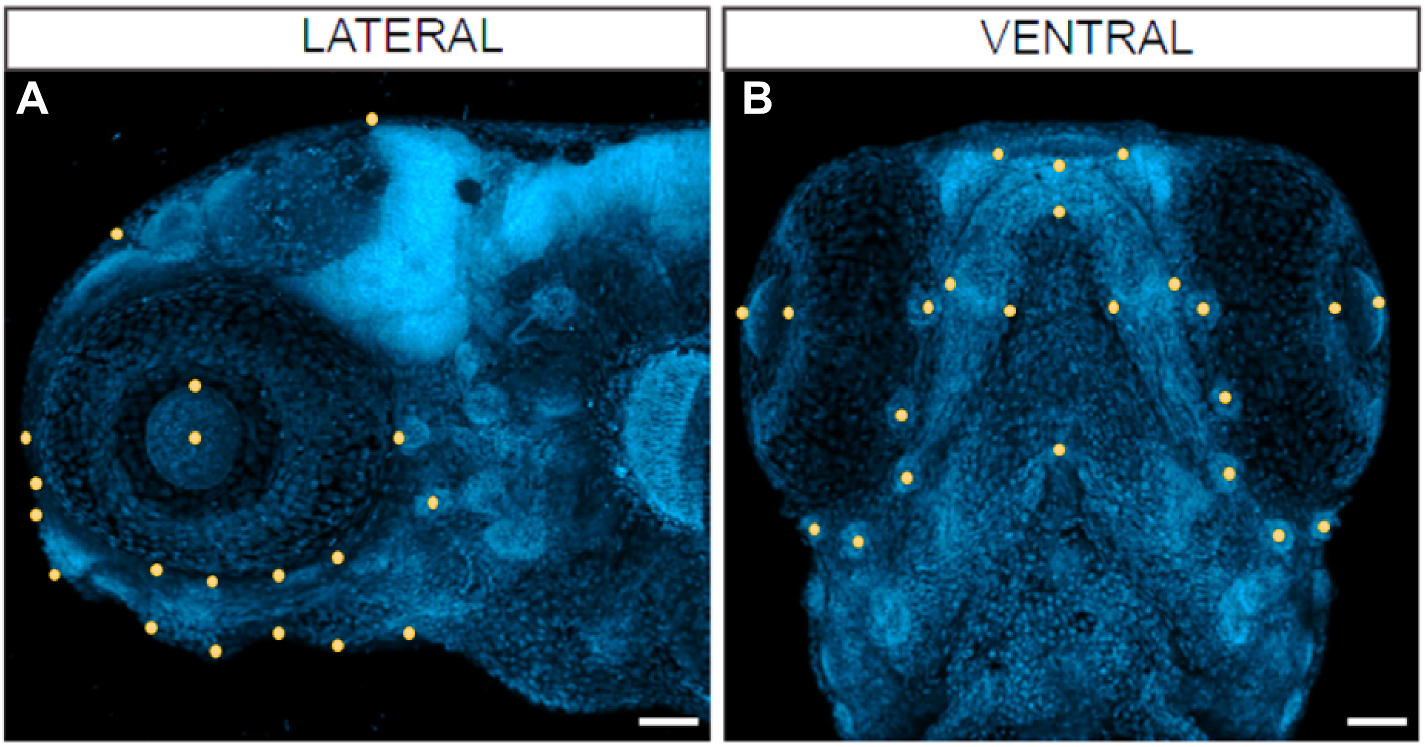

Fig. S6. Expansion of zFACE to evaluate craniofacial images in other orientations. Suggested landmark placement for the craniofacial region in a) lateral and b) ventral images at 5 dpf**.**

Disease Models & Mechanisms • Supplementary information

Table S1. zFACE landmark and measurement definitions.

Landmarks:

Landmark	Name	Definition
1	Right eye pupil	Right pupil apex point
2	Right dorsal neuromast	Center of the right neuromast on top of the olfactory placode
3	Right olfactory placode	Point in the center of the olfactory placode
4	Right middle neuromast	Center of the right neuromast below the olfactory placode
5	Right chelion	Point at the junction of the upper lip and lower lip, located on the right labial commissure
6	Midpoint from right chelion to labiale inferius	Midpoint from right chelion to labiale inferius
7	Labiale inferius	Midpoint of the lower vermillion line
8	Midpoint from left chelion to labiale inferius	Midpoint from left chelion to labiale inferius
9	Left chelion	Point at the junction of the upper lip and lower lip, located on the left labial commissure
10	Left middle neuromast	Center of left neuromast below the olfactory placode
11	Left olfactory placode	Point at the center of the left olfactory placode
12	Left dorsal neuromast	Center of the left neuromast on top of the olfactory placode
13	Left midface neuromast	Center of the left neuromast between the olfactory placodes
14	Right midface neuromast	Center of the right neuromast between the olfactory placodes
15	Dorsal midpoint of face	Located in the interhemispheric brain region
16	Supralabiale	Center of the upper border of the upper lip
17	Right christa philtri	The point on each elevated margin of the philtrum just above the vermilion line
18	Labiale superius	Midpoint on the lower border of the upper lip
19	Left christa philtri	The point on each elevated margin of the philtrum just above the vermilion line
20	Sublabiale	Center of the lower border of the lower lip
21	Right ventral neuromast	Center of the right ventral neuromast
22	Indentation on right of gnathion	Point where the gnathion meet on the right
23	Gnathion (menton)	Lowest median landmark on the lower border of the mandible (Menton: most inferior median point of the chin)
24	Indentation on left of gnathion	Point where the gnathion folds meet on the left
25	Left ventral neuromast	Center of the left ventral neuromast
26	Left eye pupil	Left pupil apex point

Disease Models & Mechanisms • Supplementary information

Measurements:

Measurement	Name	Definition
**1**	Width	Point Measure from point 1 to 26
**2**	Height	Measurement from point 15 to 23
**3**	Olfactory distance	Measurement from point 3 to 11
**4**	Upper lip width	Measurement from point 16 to point 18
**5**	Lower lip width	Measurement from point 7 to point 20
**6**	Mouth width	Measurement from point 5 to point 9
**7**	Olfactory to mouth	Angle 11 to 3 to 16
**8**	Olfactory to mouth 2	Angle 3 to 11 to 16
**9**	Olfactory difference	Difference of Olfactory to Mouth 1 and Olfactory to Mouth 2
**10**	Olfactory to mouth 3	Angle 3 to 16 to 11
**11**	Chin width	Measurement from point 21 to point 25
**12**	Mouth to chin	Measurement from point 16 to point 23
**13**	Alternate height	Midpoint from points 2 to 12 to point 23
**14**	Mouth height	Measurement from point 16 to point 20
**15**	Neuromast angle 1	Angle 2 to 4 to 16
**16**	Neuromast angle 2	Angle 12 to 10 to 16
**17**	Neuromast difference	Difference between neuromast angle 1 and neuromast angle 2
**18**	Neuromast height	Midpoint from points 2 to 12 to midpoint from points 4 to 10
**19**	Neuromast width	Midpoint from points 2 to 4 to midpoint from points 12 to 10
**20**	Mid neuromast width	Measurement from point 13 to point 14
**21**	Average length olfactory to mouth	Average measurement from points 3 to 16 and from points 11 to 16
**22**	Area top	Area from measurement of points 2 to 12 to 10 to 4
**23**	Area bottom	Area from measurement of points 4 to 10 to 25 to 21
**24**	Area combined	Sum of value from area top and area bottom
**25**	Mouth area	Sum of areas from points 5 to 17 to 18 and points 9 to 19 to 18
**26**	Mouth perimeter	Sum of measurements from points 5 to 17, 18 to 17, 19 to 18, 9 to 19, 8 to 9, 7 to 8, 6 to 7, and 5 to 6
**27**	Mid olfactory to chin height	Midpoint from points 3 to 11 to point 23
**28**	Labiale superius angle	Angle 5 to 18 to 9
**29**	Chelion left angle	Angle 5 to 9 to 18
**30**	Chelion right angle	Angle 9 to 5 to 18
**31**	Chelion difference	Difference between the left and right chelion angles
**32**	Labiale inferius angle	Angle 17 to 7 to 19
**33**	Christa philtri left angle	Angle 17 to 19 to 7
**34**	Christa philtri right angle	Angle 19 to 17 to 7
**35**	Christa philtri difference	Difference between left and right christa philtri angles
**36**	Labiale superius mid angle	Angle 6 to 18 to 8
**37**	Labiale inferius left angle	Angle 6 to 8 to 18
**38**	Labiale inferius right angle	Angle 8 to 6 to 18
**39**	Labiale inferius difference	Difference between the Labiale Inferius Left Angle and Labiale Inferius Right Angle

Disease Models & Mechanisms • Supplementary information

Table S2. Component loadings of zFACE measurements after promax rotation for PCA analysis of development.

Variable	PC1	PC2	PC3	PC4	PC5	PC6	Unexplained
Width							0.11
Height							0.14
Olfactory Distance				0.51			0.12
Upper Lip Width						-0.38	0.37
Lower Lip Width							0.53
Mouth Width							0.07
Olfactory to Mouth							0.04
Olfactory to Mouth 2							0.05
Difference					0.30		0.48
Olfactory to Mouth 3							0.04
Chin Width							0.15
Mouth to Chin							0.03
Alternate Height							0.10
**Mouth Height**		0.41					0.05
Neuromast Angle 1							0.11
Neuromast Angle 2							0.16
Difference						0.77	0.25
Neuromast Height							0.10
**Neuromast Width**	0.32						0.10
Mid Neuromast Width				0.38			0.24
Average Length Olfactory to mouth				0.47			0.05
Area Top				0.36			0.10
Area Bottom							0.04
Area Combined							0.06
Mid Olfactory to Chin height							0.10
**Mouth Area**		0.32					0.13
Mouth Perimeter							0.05
Libiale Superius Angle			-0.42				0.10
Chelion Left Angle			0.45				0.13
Chelion Right Angle			0.36				0.21
Chelion Diff					0.70		0.31
**Labiale Inferius Angle**		-0.33					0.03
**Crista Philtri Left Angle**		0.33					0.03
**Crista Philtri Right Angle**		0.32					0.05
Crista Philtri Diff							0.24
Labiale Superius Mid Angle							0.02
Labiale Inferius Left Angle							0.09
Labiale Inferius Right Angle							0.06
Labiale Inferius Diff							0.28

Disease Models & Mechanisms • Supplementary information
